# COVID-19 vaccination is not associated with reduced SGA or low Apgar score

**DOI:** 10.1038/s41390-023-02875-w

**Published:** 2023-10-31

**Authors:** Amr Ehab El-Qushayri

**Affiliations:** Faculty of Medicine, Minia University, Minia, Egypt

Dear Editor,

Since the introduction of COVID-19 vaccines, substantial improvement has been noticed regarding severity, hospital and ICU admission and mortality of COVID-19.^1^ Per se, the clinical society started to test the different types of vaccines on pregnant women, and it is safety was measured by pregnancy outcomes.

The article of Zhang et al.^2^ demonstrated that vaccinated pregnant women were associated with a significant reduction of small gestational age (SGA) and low Apgar score at 5 min (<7) prevalence. Despite the fact that the authors included all the reported evidence regarding the safety of COVID-19 vaccination during pregnancy, the paper had some methodological and statistical concerns that progressed to results’ bias.

**Fig. 1:**
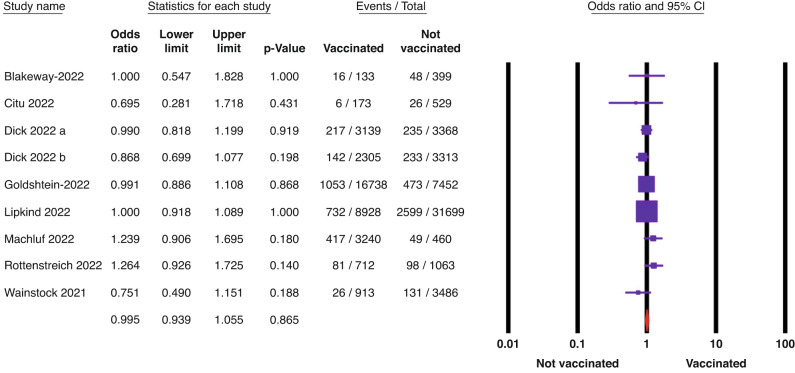
The association between SGA and COVID-19 vaccination represented by the odds ratio (OR) and the 95% confidence interval (95% CI).

**Fig. 2:**
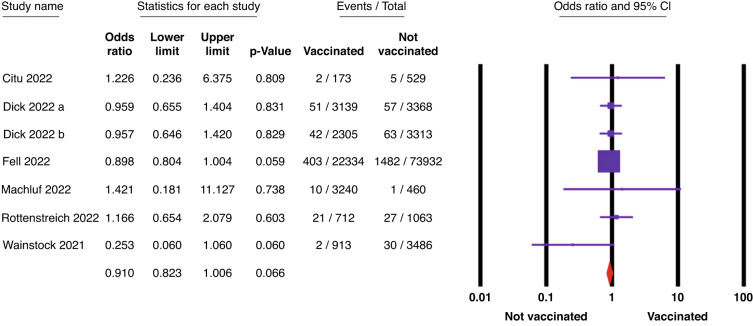
The association between low Apgar score at 5 min (<7) and COVID-19 vaccination represented by the odds ratio (OR) and the 95% confidence interval (95% CI).

First, the significance of the two outcomes, SGA and low Apgar score at 5 min (<7), was driven by only one paper (Magnus et al. 2022),^3^ which weighted 61.3% and 47.3% in both outcomes, respectively. Therefore, by performing sensitivity analysis by removing the largest weight study (Magnus et al. 2022), the significance of both outcomes was lost (odds ratio (OR): 1, 95% confidence interval (CI): 0.95–1.05, *p* = 0.87) and (OR: 0.91, 95% CI: 0.82–1.01, *p* = 0.07) for SGA and low Apgar score at 5 min (<7), respectively (Figs. 1 and 2). Fixed effect model was used due to the absence of significant heterogeneity (*p* > 0.05).

Second, the authors included two duplicate papers including similar populations, Goldshtein et al. (2021)^4^ and Goldshtein et al. (2022).^5^ The two studies were conducted in the same country, had nearly similar authors, and in both studies, women received BNT162b2 vaccination. Moreover, the study of Goldshtein et al. (2021) included pregnant women who were vaccinated from December 19, 2020, till February 28, 2021, while the Goldshtein et al. (2022) included all singleton live births from March 1, 2021, till September 31, 2021, which means that their mothers were pregnant at least 7 months before such date and this observation increases the possibility that both populations were the same.

Despite the fact that COVID-19 vaccines exhibited beneficial efficacy outcomes, they were not associated with a reduction of SGA and low Apgar score at 5 min (<7). More studies—in particular randomized ones—are still needed to confirm if COVID-19 vaccines are associated with a high safety margin and could significantly prevent adverse pregnancy outcomes.
